# Chemical profile and in vitro antischistosomal activity of *Crotalaria madurensis* different extracts

**DOI:** 10.1038/s41598-026-60749-7

**Published:** 2026-07-10

**Authors:** Mona A. Mohamed, Samia William, Mosad A. Ghareeb, Sanaa S. Botros

**Affiliations:** 1 Medicinal Chemistry Department, Theodor Bilharz Research Institute, Kornaish El-Nile, Warrak El-Hadar, P.O. 30), Imbaba, Giza, 12411 Egypt; 2 Parasitology Department, Theodor Bilharz Research Institute, Kornaish El- Nile, Kornaish El-Nile, Warrak El-Hadar, P.O. 30), Imbaba, Giza, 12411 Egypt; 3 Pharmacology Department, Theodor Bilharz Research Institute, Kornaish El-Nile, Warrak El-Hadar, (P.O. 30), Imbaba, Giza, 12411 Egypt

**Keywords:** *Crotalaria madurensis*, Antischistosomal, Triterpenes, Quercetin, Cinnamic acid derivatives, Good health and well-being, Biochemistry, Chemical biology, Chemistry, Drug discovery, Plant sciences

## Abstract

Chromatographic isolation of *Crotalaria madurensis* Wight & Arn leaves aqueous methanol extract availed a new potassium sulfonate acylated flavonol glycosides named quercetin 8-potassium sulfonate 3-*O*-[2-*O*-sulfonyl]-*β*-**D**-^4^C_1_-glucopyranosyl-(1’’’’→3’’’)-4-*O*-[*E*- caffeoyl]-*β*-**D**-^4^C_1_-glucopyranosyl-(1’’’→2’’)-3-*O*-[*E*-caffeoyl]-*β*-**D**-^4^C_1_-glucopyranoside (*Crotalamadoside* A) (**1**), along with three known flavonoids identified as quercetin 7-*O*-neohispredoside (**2**), 3’,4’-dimethoxy quercetin 3-*O*-neohispredoside (**3**) and isoquercetin (**6**) besides two triterpene saponin compounds named as hedragenin 3-*O*-*β*-**D**-^4^C_1_-glucopyranoside (**4**) and hedragenin 3-*O*-*α*-**L**-^1^C_4−_rhamnopyranoside (**5**). This was in addition, to two cinnamic acid derivatives named as *E*-caffeic acid 4-*O*-*β*-**D**-^4^C_1−_glucopranoside (**7**) and *E*-caffeic acid (**8**). Identification of the isolated compounds relied on their chromatographic properties and spectral data (UV, ESI-MS, ^1^H NMR, ^13^C NMR, ^1^H-^1^H COSY, HSQC, and HMBC). The antischistosomal activity of the *C. madurensis* different extracts were assessed in vitro using schistosome worm killing, findings revealed that the aqueous methanol extract was the most effective extract, chromatographic fractionation of this extract revealed significant antischistosomal activity for fractions number V and VI rich in flavonoids and triterpene saponins, respectively.

**Supplementary Information:**

The online version contains supplementary material available at 10.1038/s41598-026-60749-7.

## Introduction

The genus *Crotalaria*, belonging to the family Fabaceae is a large family of plants containing over 700 species, mainly found in Mexico, Brazil and India, as well as East and South Tropical Africa. They were used traditionally in veterinary medicine^1^. The ornamental shrub *Crotalaria madurensis* Wight & Arn is native in India Madura hills and Nilgiris^2^. *C. madurensis*, demonstrated several biological activities including antimicrobial, antioxidant^3,4^, anti-inflammatory and antischistosomal activities^3^, besides analgesic and gastric protective properties^5^. Phytochemical investigation of *C. madurensis* revealed flavonoids^4,3,5^, triterpene saponins in addition to myo-inositol^3^.

Also, a large number of secondary metabolites were identified from the genus *Crotalaria*, these included several types of alkaloids^6–9^ particularly pyrrolizidine alkaloids^10–12^, in addition to flavonoids^4,3,13^, tannins^14,7,15^, phenolic compounds^13,16,10^, chalcones^17,18^, coumarins^19^, chromenes^20^, cyclopentylidene^21^, steroids^14,7^, triterpenes^13,7,3^, terpenoids^14,15^, phenylpropanoids^22,23^, heterodimer^24^, polysaccharides^13,7,25^. Besides primary metabolites, like protein^26,27^, fatty acids^28^, fatty alcohol (1-nonadecanol)^29^, and carbohydrates^7,30^ were also identified as well as volatile oils^26^.

Genus *Crotalaria* have antimicrobial activity^4,6,3^, anti-inflammatory^3,31,32^, antiparasitic (e.g. nematicidal^33^, leishmanicidal^34^ antimalarial^17,35^, antischistosomal^3^, molluscicidal^36^ and anthelmintic activities^37^. In addition, to antioxidant^3,31,14,13,38^, cytotoxic^13,38,31^, anticancer^19^ and hepatoprotective activities^39,40^, etc. Additional, evaluation of the ethnobotanical uses of this genus with prominent medical significance showing diverse activity ranges against many illnesses will help in further understanding of its therapeutic potential, enabling its further use in the treatment of various diseases.

The ongoing study focuses on the chromatographic separation and structural identification of a novel acylated flavonol glucosides 8-sulfonate, three flavonol compounds, two triterpenoidal saponins and two cinnamic acid derivatives from the leaves of *C. madurensis*. In this work, search for effective bioactive components against schistosomiasis was targeted by the primary investigation of study *C. madurensis* leaves different extracts as well as fractions resulting from column chromatographic separation of the most promising active extract. *C. madurensis* antischistosomal potential has been primarily investigated in vitro using schistosome worm killing to complement on previous findings reported by the authors of this study^3,41–44^, highlighting the potential of local Egyptian plants as a source of natural bioactive products that may prove to be a promising in the treatment of one of the detrimental trematodal diseases. This research is directly aligned with Sustainable Development Goal 3 (Good Health and Well-being), contributing to global efforts aimed at improving health outcomes, preventing disease, and promoting overall well-being.

## Materials and methods

### Plant material

*Crotalaria madurensis* Wight & Arn is a cultivated plant; its leaves were collected from Al-Kanater Al-Khiraya, Kaliobia, Egypt (30.1841° N, 31.1803° E) during April 2022, following relevant international guidelines and legislation, with all necessary permissions obtained. The collection process adhered to standard practices for plant collection and preservation. The plant was identified by Prof. Wafaa M. Amer, Botany Department, Faculty of Science, Cairo University, Giza, Egypt. A voucher specimen (Reg. No. CM-II/2022) was deposited at the herbarium of the Medicinal Chemistry Department, Theodor Bilharz Research Institute, Giza, Egypt, which serves as a repository for authentic plant specimens and ensures their long-term preservation for future reference. Identification involved comparing plant features with recognized taxonomic references and expert knowledge in plant taxonomy. This study was conducted in full compliance with the IUCN Policy Statement on Research Involving Species at Risk of Extinction and the Convention on the Trade in Endangered Species of Wild Fauna and Flora (CITES). No endangered or protected species were involved, and all applicable national and institutional regulations were strictly followed.

### Equipments

The NMR spectra were measured at frequencies of 500 or 300 (^1^H) and 125 or 75 (^13^C) MHz using a JEOL GX-500 spectrometer or a Varian Mercury 300 NMR spectrometer respectively, and *δ* in ppm unit values were reported relative to TMS in pyridine-*d*_5_ or DMSO-*d*_6_. Analyses of ESI-MS were performed on a LTQ-FT-MS spectrometer (Thermo Fisher Scientific, Bremen, Germany).

### Chromatographic material

For chromatographic separation, microcrystalline cellulose S (Merck, Darmstadt, Germany), Sephadex LH-20 (Pharmacia, Uppsala, Sweden), polyamide 6 S (Riedel de Haën AG, Seelze, Germany) and silica gel G 60 powder (Merck, Germany) were used for column chromatography (CC). The detection of compounds was based on comparative and 2D-paper, using Whatman No. 1 paper chromatography (PC) (Whatman, UK). The triterpene compounds homogeneity were tested using thin layer chromatography (TLC) F254 plates (Merck, Germany), and the spots were visualized by spraying with H_2_SO_4_ (20% in MeOH) as spray reagent and then heating for 5 min at 120 °C.

### Extraction and isolation

*C. madurensis* powdered, dried leaves (900 g) were exhaustively extracted under reflux (70 °C) with 70% methanol (5 × 6 L). Then the methanol was removed and the salts were removed from the resulting concentrated 70% extract by adding an excess of ethanol to precipitate the dry residue (175 g). Fat was removed from the extract through reheating with petroleum ether (60–80 °C). Defatting and desalting dry extract obtained (150 g) was suspended in H_2_O and subjected to fractionation using a polyamide column (Ø 5.0 × 150 cm, 500 g) which was eluted with H_2_O and then with H_2_O-MeOH gradients ratios until achieving the 100% MeOH. Depending on the changes in the R_*f*_–values shown by the analyses of TLC sprayed with 20% H_2_SO_4,_ followed by heating at 120 °C, along with PC assisted by long UV light 365 nm and Naturstoff as spray reagent, a total of 50 individual samples (each 750 mL) were collected in 9 collective fractions (I-IX). There were no compounds in fractions I to IV eluted with H_2_O, followed by 5–10% MeOH, but there were brown viscous material, mostly of free sugars. Fr. V (20% MeOH, 27 g) was dissolved in methanol and a crude sample of the main compound **1** was spontaneously precipitated using the excess acetone. The precipitate 650 mg was then purified on a Sephadex LH-20 column (Ø 2.0 × 25 cm), using methanol to obtain pure compoumd **1** (70 mg). The remaining mother liquor after precipitation was separated over Sephadex LH-20 column chromatography, using MeOH: H_2_O, for elution (60:40%) to obtain pure compound **2** (25 mg) and **3** (20 mg). Fr. VI (40%, MeOH, 26 g), contained a mixture of the main saponin compounds (4 and 5), which was dissolved in ethanol and was precipitated by the addition of 40% ethyl ether (v/v), followed by separation on silica gel C eluted with 60:40% MeOH-CH_2_Cl_2_ (v/v), resulting in samples of the two crude compounds, which were then purified on Sephadex LH-20 using ethanol to obtain pure compoumd **4** (25 mg) and compoumd **5** (39 mg). Fr. VII (60% MeOH, 25 g) contained a mixture of saponin and flavonoid compounds. The dried fraction was dissolved in H_2_O, then filtered and extracted using EtOAc (17 g) followed by *n*-BuOH (3 g). The flavonoid compounds were mainly found in the ethyl acetate extract while saponin compounds were mainly extracted by *n*-butanol. EtOAc extract fractionated via Sephadex LH-20 column with aqueous ethanol (40% EtOH) as an elution solvent, and then by a cellulose column eluted with BIW (*n*-BuOH/2-propanoI/H_2_O, 4:1:5 v/v/v, organic layer) to obtain a pure compoumd **6** (35 mg). Fraction VIII (80% MeOH, 17 g) underwent repeated chromatographic separation on cellulose followed by Sephadex LH-20 columns, using 20–60% (H_2_O: MeOH) as an eluent, to get a pure compoumd **7** (35 mg). Fraction IX (MeOH, 19 g) a pure compoumd **8** (30 mg) was obtained from this fraction after separation over Sephadex LH-20 eluted with EtOH.

All isolation steps were monitored with the aid of co-TLC using the following solvent systems: MeOH-CH_2_Cl_2_ (1.5:8.5), EtOAc-CH_2_Cl_2_ (7.5:2.5), MeOH-EtOAc-CH_2_Cl_2_-H_2_O (30:33:32:5) and *n*-BuOH-EtOH-H_2_O (65:20:15) or for flavonoids 2D-PC using, S1: *n*-BuOH-HOAc-H_2_O (4:1:5, organic phase) and S2: 15% acetic acid in H_2_O.

### **Detection of sulfate group in compound** (Crotalamadoside A) **(1)**

For 2 h compound **1** (5 mg) was heated with 2 N HCl in water bath and neutralized with NaHCO_3_. The solution was then dried, and the residue underwent paper chromatography using EtOH, followed by air-dried, and sprayed with solution of BaCl_2_. Next the paper spray with solution from potassium rhodizoate after it has air-dried. The presence of sulfate groups was indicated by the resulting of yellow color^45^.

### Preparation of tested antischistosomal extracts

About 100 g of *C. madurensis* leaf powder was extracted separately using CH_2_Cl_2_, EtOAc, and *n*-butanol. The previous extracts were evaporated to produce extracts (**A**), (**B**), and (**C**), respectively. About 100 g of *C. madurensis* leaf powder extracted with 70% methanol that was evaporated resulting in an aqueous extract which was defatted using petroleum ether and then precipitated using additional ethanol to remove the salts; yielding the extract (**D**).

### Preparations of plant extracts/ fractions and Praziquantel (PZQ)

CH_2_Cl_2_, EtOAc, *n*-butanol and crude methanol extracts along with its fractions were freshly prepared before use as stock solutions of 1 mg/ml in MeOH and diluted with distilled H_2_O. The reference drug PZQ as well, was freshly prepared before use as stock solutions of 1 mg/mL in DMSO.

### Potential antischistosomal activity of *C. madurensis* using in vitro Schistosome worm killing

Worms were obtained from the Schistosome Biological Supply Center (SBSC), TBRI. They infected each Syrian golden hamster (*Mesocrietus auratus*) by 350 *Schistosoma mansoni* cercariae^46^. worms collected in small petri-dishes containing RPMI 1640 media (plus glutamine, 20% newborn calf serum and antibiotics (streptomycin, penicillin and gentamicin) and kept in a CO_2_–incubator at 37 °C. After preparation of different plant samples concentrations to be tested, duplicate experiment was used for each concentration/ plant samples and worms in an average number of 8–10 were placed in new clean dishes with the aid of Pasteur pipette. Residual media was decanted and fresh media (3 mL/dish) with the desired concentrations of test *C. madurensis* samples were placed in each plate. Only pure medium was used for -ve control, medium with methanol or dimethyl sulfoxide and media of +ve control containing praziquantel were simultaneously used. After an overnight incubation in CO_2_-incubator, the new media containing the test plant extract was decanted and worms were placed in sterile saline and then the dishes were placed in CO_2_- incubator. Saline was then removed and 2 mL fresh media was added before placing dishes back into the CO_2_-incubator. The movement of worms was noticed, on the second day and the media was changed again. The dishes were left for two more days and on the 5th day, the ratio of the living to dead worms was estimated. After the observation period was ended (5 days), the worms in a laminar flow hood were examined for viability with the help of stereomicroscope. The final worm mortality % was record by calculating the ratio between dead worm numbers and total worm numbers.

## Results and discussion

### Phytochemical analysis and spectroscopic data of known compounds along with structure’s elucidation of compound (1)

**Fig. 1:**
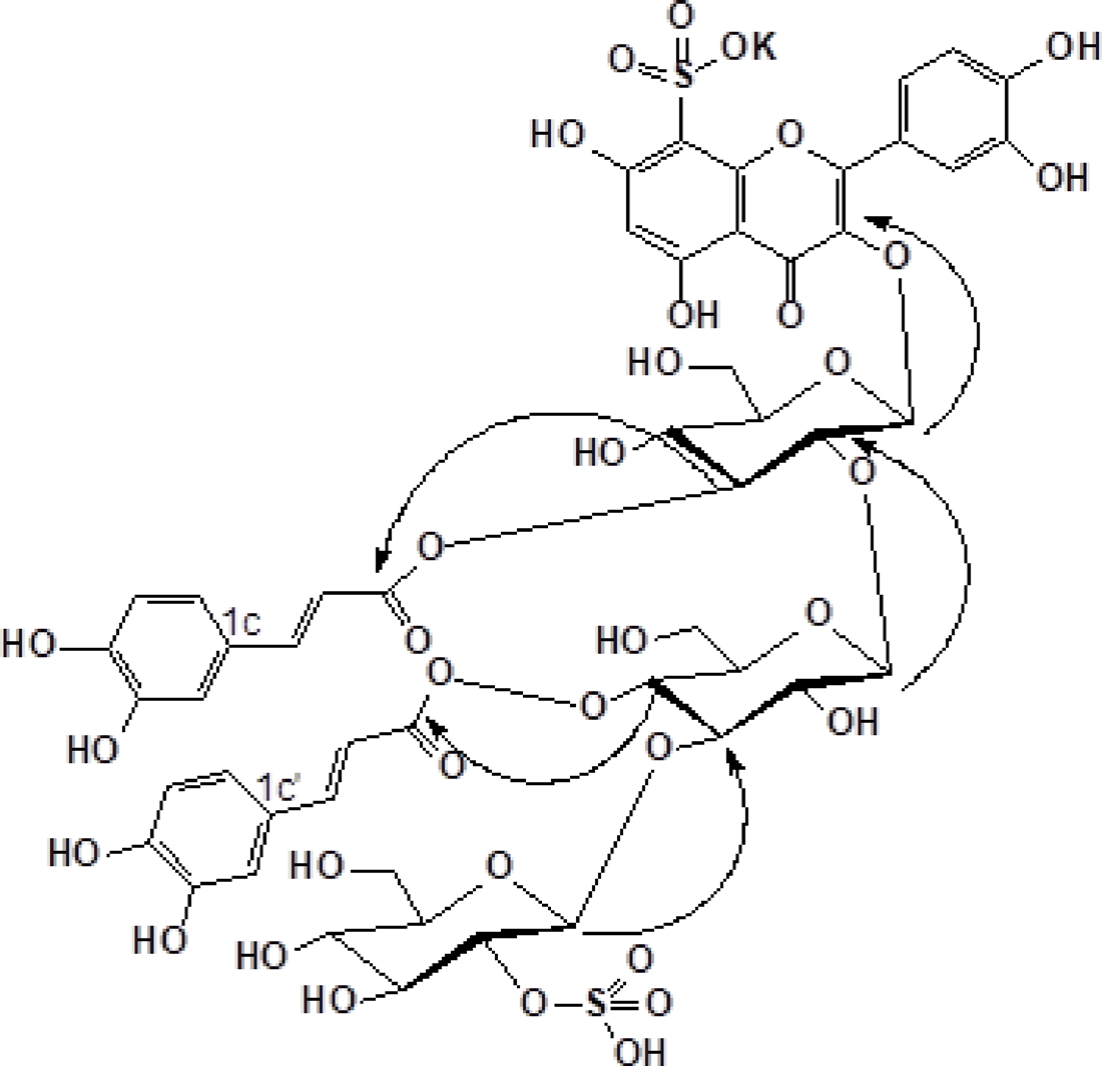
Selected HMBC correlations for compound **1**. Arrows point from H to C atoms.

**Fig. 2:**
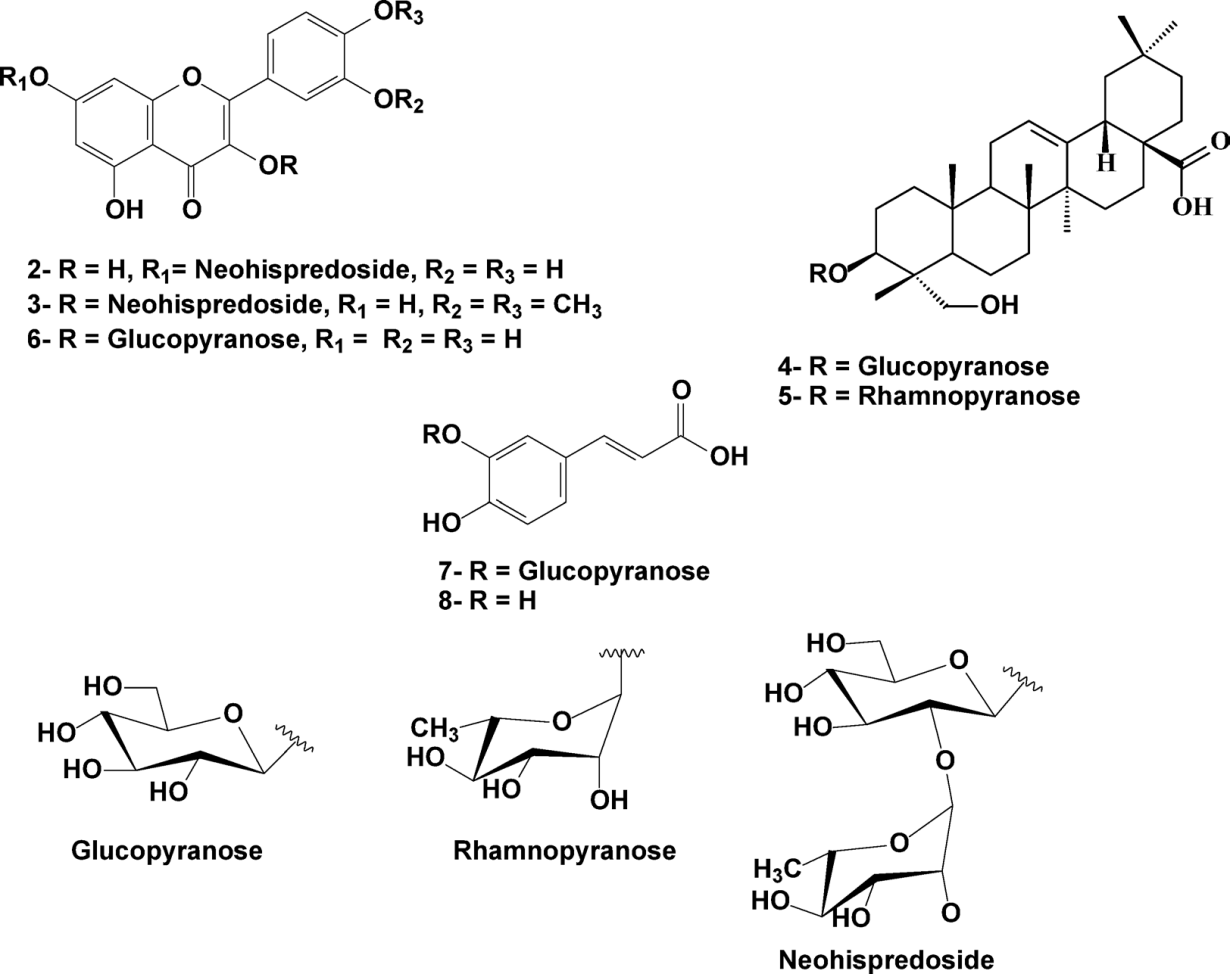
Chemical structures of the isolated compounds.

Phytochemical isolation of *C. madurensis* led to a new quercetin 8-potassium sulfonate 3-*O*-[2-*O*-sulfonyl]-*β*-**D**-^4^C_1_-glucopyranosyl-(1’’’’→3’’’)-4-*O*-[*E*-caffeoyl]-*β*-**D**-^4^C_1_-glucopyranosyl-(1’’’→2’’)-3-*O*-[*E*-caffeoyl]- *β*-**D**-^4^C_1_-glucopyranoside (1), besides seven known compounds (**2–8**) (Figs. 1 and 2). The structures of the known compounds were determined by comparing their spectral data with those previously reports and were confirmed by co-chromatography with authentic samples. The known flavonol compounds were identified as quercetin 7-*O*-neohispredoside (**2**), 3’,4’-dimethoxy quercetin 3-*O*-neohispredoside (3)^47,48^, and isoquercetin (**6**)^49^. The triterpene compounds were identified as hedragenin 3-*O*-*β*-**D**-^4^C_1_-glucopyranoside (**4**)^50^ and hedragenin 3-*O*-*α*-**L**-^1^C_4−_rhamnopyranoside (**5**)^50^. This was in addition to the cinnamic acid derivatives, that were identified as *E*-caffeic acid 4-*O*-*β*-**D**-^4^C_1−_ glucopyranoside (**7**)^51^ and *E*-caffeic acid (**8**)^52^.

Based on chromatographic characters, UV spectra and the results of acid hydrolysis, compound **1**, was considered as a acyl flavonol glucosides, based on its UV spectrum (in MeOH) which showed a specific band I at λ_max_ ≈ 319 nm. In addition to a band II being blocked by an additional strong maximum at 263, 275 nm, approving the flavonol structure associated with diacyl moieties^53^. Acid hydrolysis of compound 1 afforded glucose in the aqueous phase as well as caffeic acid and new aglycone that were detected in the organic phase (Comparing TLC and PC with authentic samples).

The ESI-MS –ve and +ve ion modes of compound 1 showed a molecular ion peaks at *m/z* 1308.78 [M-H]^–^ and 1311.279 [M + H]^+^, respectively, which corresponds to 1310 molecular weight and molecular formula of C_51_H_51_KO_34_S_2_. In the ESI-MS +ve ion mode, the fragment ion peak at 1215.106 = [M+H-SO_4_]^+^, represents a lossof 96 mass units. There are five diagnostic fragment ion peaks at *m/z* 1131.355 [M + H-deoxycaffeoyl-H_2_O]^+^, *m*/*z* 987.281 [M + H*–*2deoxycaffeoyl]^+^, *m*/*z* 744.831 [M + H-2deoxycaffeoyl-(deoxyglucose+deoxsulfate)]^+^, *m*/*z* 583.284 [M + H-2deoxycaffeoyl-(deoxyglucose+deoxsulfate)-deoxyglucose]^+^ and 421.429 [M + H-2deoxycaffeoyl- (deoxyglucose+deoxsulfate])-2deoxyglucose]^+^ = [aglycone + H]^+^, which indicating that the aglycone M.W is 420 [quercetin+KSO_3_]. ^1^H-NMR spectrum of compound 1 (Table 1) exhibited an ABX spin coupling system for benzene-ring trisubstituted in 1’, 3’,4’-positions consists of signals at δ_H_ 7.89 (1H, *brs*), 7.82 (1H, *brd*, *J* = 8.4 Hz) and 6.83 ppm (1H, *d*, *J* = 8.4 Hz) assigned to H-2’, H-6’ and H-5’, respectively. The singlet signal at δ_H_ 6.11 was assignable to H-6 in trisubstituted A-ring. The substitution of C-8 was confirmed by its downfield shift to δ_C_ 99.1. Thus, the aglycone moiety permitted the establishment of quercetin 8-potassium sulfonate by comparison of its NMR data as well as mass fragmentation result with structurally related compounds^54,55^. The existence of two caffeoly units in compound 1 was deduced through the detection of two groups of type A_2_ × _2_ olefinic resonances with (*E*) configuration identified through the large coupling constant and both integrated for two proton signals at δ_H_ 7.17, 5.92, 7.41 and 6.22 ppm, each of which (1H, *d*, *J* = 16.05 Hz) for H-7c and H-8c as well as H-7c’ and H-8c’, respectively. This was besides two ABX spin coupling systems each consisted of three proton signals at δ_H_ 6.89 (1H, *brs*), 6.85 (1H, *brd*, *J* = 9.9 Hz) and 6.68 ppm (1H, *d*, *J* = 7.65 Hz), assignable for H-2c, H-6c and H-5c, respectively as well as δ_H_ 6.98 (1H, *brs*, 6.92 (1H, *brd*, *J* = 7.65 Hz) and 6.71 ppm (1H, *d*, *J* = 8.4Hz) assignable for H-2c’, H-6c’ and H-5c’, respectively. The^1^ H-NMR spectrum exhibited three anomeric proton signals at δ_H_ 5.74, 4.29, and 4.21 ppm, each (*d*, *J* = 7.6 Hz), which were directly coupled through one- bond in HSQC spectrum with their corresponding carbon signals at δ_C_ 102.4, 103.4 and 104.1 ppm, respectively. The polysaccharides units were concluded to have *β*–**D**-^4^C_1_-glucopyranosyl stereo structure, depending on acid hydrolyses, the large *J* values of their distinct anomeric protons^56^ and their^13^ C-resonances δ values besides large *J* values showed for an oxymethine protons at δ_H_ 3.13–5.46 ppm thus indicated their axial–axial coupling distinctive for glucopyranosyls (Table 1). The^13^ C-NMR resonances exhibited 51 signals, 15 signals were belonging to aglycone moiety, 18 for two caffeoyl moieties and 18 for three *β*-**D**-glucopyranosyl moieties. The binding site of the carbohydrate units was revealed to be C-3 of aglycone due to the upfield location of C-3 signal (≈ 2.1 ppm) along with the location of C-2 was downfield clearly (≈ 10 ppm) in comparison to its corresponding carbons signals in the unsubstituted quercetin^57^.

Findings were further substantiated by the HMBC spectrum which displayed long-range correlation between H-1’’ glucose signal at δ_H_ 5.74 ppm and the C-3 signal of aglycone at δ_C_ 132.9 ppm. The interglucosidic linkages were deduced from the clearly shielded location of the C-2’’ signal in inner glucopyranose at δ_C_ 82.3 (≈ + ∆ 9.5 ppm) compared to its corresponding^13^ C signal location of the terminal glucopyranose δ_C_ (72.9)^58^. This further enhances verification of long range three bond HMBC correlations which showed correlations between H-1’’’ (4.29 ppm) glucosyl and C-2’’ glucosyl (82.3 ppm). Similarly, correlations were shown between H-1’’’’ (4.21 ppm) of the external glucosyl and C-3’’’ (85.9 ppm) to create a triglucosyl moieties at C-3 as 3-*O*-*β*-**D**-^4^C_1_-glucopyranosyl (1’’’’→3’’’)-*O*-*β*-**D**-^4^C_1_-glucopyranosyl (1’’’→2’’)-*O*-*β*-**D**-^4^C_1_-glucopyranoside. The attachment of the sulfate group to H-2’’’’ of outer glucose, was inferred from the downfield shift of H-2’’’’ to δ_H_ 4.91 ppm as a result of sulfation shift that was further confirmed from^13^ C spectrum by the effect of the alpha shift of the glucose C-2’’’’ signal to δ_C_ 83.9 ppm, instead of the approximately δ_C_ 73.0 ppm showed in the non-sulfated glucose. The binding sites of the two acyl moieties at both 3’’-OH and 4’’’-OH were deduced from distinct downfield shift of its H-3’’ and H-4’’’ at δ_H_ (5.46 ppm) and (5.13 ppm), respectively. This was beside the slight downfield shift of both C-3’’ and C-4’’’ revealed from^13^ C-resonances relative to the free glucopyranosyl analog. This was evidenced from the HMBC spectrum which exhibited three bond correlations between the ester carbonyl carbon signal at δ_C_ 165.8 ppm (C-9) and H-3’’ at δ_H_ 5.46 ppm as well as the other ester carbonyl carbon signal at δ_C_ 166.1 ppm (C-9’) and H-4’’’ at δ_H_ 5.13 ppm. The remaining^1^ H and^13^ C-signals of the three monosaccharides and caffeoyl units were also identified with the help of the interpretation of the^1^ H-^1^H-COSY, HSQC and HMBC-correlation peaks (Table 1). This as in addition to comparison with the corresponding reported data on the acylated and sulfonated flavonol sulfated glucosides related compounds^59–63^ to identify the complete structure as that of quercetin 8-potassium sulfonate 3-*O*-[2-*O*-sulfonyl]-*β*-**D**-^4^C_1_-glucopyranosyl-(1’’’’→3’’’)-4-*O*-[*E*-caffeoyl]-*β*-**D**-^4^C_1_-glucopyranosyl-(1’’’→2’’)-3-*O*-[*E*-caffeoyl]-*β*-**D**-^4^C_1_-glucopyranoside.

To the best of our knowledge, in this study compound 1 aglycone (querecetien-8-potassium sulfonate) was isolated for the first time in nature; although Woz´nicka et al. had synthesized quercetin-8-sulfonic acid and its sulfonated sodium salt previously^55^. Although sulfated flavonoids containing C-O-SO_3_-linkage are known to exist in various plant species, sulfonated flavonoids that have C-S-O_3_-linkage are recorded to be of rare incidence and found in compounds such as sodium quercetin-5ˈ-sulfonate and sodium apigenin-3ˈ-sulfonate^64,65^. The first known examples of 8-sulfonated flavonoids from a natural origin are galangin-8-sulfonate and its 3-*O*-glucoside besides kaempferol-8-sulfonat^66^.

**Table 1:** ^1^H, ^13^C-NMR and HMBC data (500/125 MHz, DMSO-*d6*) of (*Crotalamad*oside A) (1).

No.	δ_C_	δ_H_ mult. ( J)	HMBC
2	156.8		
3	132.9		
4	177.8		
5	161.0		
6	98.1	6.11*s*	C-8,10
7	162.6		
8	99.1		
9	156.1		
10	104.8		
1’	121. 8		
2’	115.3	7.89 *brs*	C-4’,6’
3’	145.1		
4’	148.7		
5’	116.4	6.83*d* (8.4)	C-1’, 3’
6’	121.8	7.82 *brd* (8.4 )	C-2’, 4’
1’’	102.4	5.74*d* (7.6)	C-3, 3’’
2’’	82.3	3.68 dd*	C-1’’’, 4’’
3’’	77.4	5.46 *t* (10.3)	C-1’’, 5’’, 9c
4’’	70.3	3.01 *t*, *	C-2’’, 6’’
5’’	77.0	3.18 *m*	C-3’’
6’’	61.1	3.50 *	C-4’’
1’’’	103.4	4.29 *d* (7.6)	C-2’’, 3’’’
2’’’	72.7	3.52 dd*	C-4’’’,
3’’’	85.9	3.83 *t*, *	C-1’’’, 1’’’’, 5’’’
4’’’	73.8	5.13 *t* (9.9)	C-2’’’, 6’’’, 9c’
5’’’	76.8	3.13*m*	C-3’’’
6’’’	61.2	3.38*	C-4’’’
1’’’’	104.1	4.21*d* (7.6)	C-3’’’’, 3’’’
2’’’’	83.9	4.91 *dd* (9.9,*)	C-4’’’’
3’’’’	75.4	3.66 *t*,(*)	C-1’’’’, 5’’’’, 1’’’
4’’’’	70.5	3.00 *t*,(*)	C-2’’’’, 6’’’’
5’’’’	77.3	3.03, *m*	C-3’’’’
6’’’’	61.6	3.31*dd* (*)	C-4’’’’
Caffeoyl at (C3’’- Glu 1)			
1c	126.1		
2c	115.8	6.89 *brs*	C-6c, 4c,7c
3c	146.1		
4c	148.7		
5c	116.3	6.68 *d* (7.65)	C-1c, 3c
6c	122.3	6.85 *brd* (9.9)	C-2c, 4c, 7c
7c	146.0	7.17 *d* (16.05)	C-2c, 5c, 9c
8c	114.2	5.92, *d* (16.05)	C-1c
9c	165.8		
Caffeoyl at (C4’’’- GluII)			
1c’	126.2		
2c’	116.2	6.98 *brs*	C-6c’, 4c’, 7c’
3c’	146.1		
4c’	149.1		
5c’	117.4	6.71, *d* (8.4)	C-3c’, 1c’
6c’	122. 8	6.92 *brd* (7.65)	C-2c’, 4c’, 7c’
7c’	145.6	7.41 *d* (16.05)	C-2c’, 5c’, 9c’
8c’	114.7	6.22, *d* (16.05)	C-1c’
9c’	166.1		

*Unresolved^1^ H signals, *δ* in ppm and *J* values in (Hz). All^13^ C and^1^ H signals were detected on the basis of 2D (^1^H-^1^H COSY, HSQC and HMBC).

Quercetin 7-*O*-neohispredoside (**2**): Off-white amorphous powder, gives yellow spot under long UV λ _max_ 365 turned to orange with Naturstoff spray reagent. Negative ESI-MS: *m/z* = 609.15 [M–H]^–^, 463.09 [M–H–rahmnose]^–^, 301.03 [M–rahmnose–glucose]^–^ = [aglycone–H]^–^. ^1^H NMR (300 MHz, DMSO-*d*_6_): *δ* = 7.57 (1H, dd, *J* = 8.4, 2.1 Hz, H-6’), 7.52 (1H, d, *J* = 2.1 Hz, H-2’), 6.82 (1H, d, *J* = 8.4 Hz, H-5’), 6.43 (1H, brs, H-8), 6.16 (1H, brs, H-6), 5.05 (1H, d, *J* = 7.5 Hz, H-1’’), 4.22 (1H, d, *J* = 7.8 Hz, H-1’’’), 3.73-3.09- (remaining sugar protons), and 0.77 (3 H, d, *J* = 6, H-6’’’). ^13^C NMR (75 MHz, DMSO-*d*_6_): *δ* = 177.4 (C-4), 163.3 (C-7), 161.2 (C-5), 156.4 (C-2), 156.2 (C-9,) 145.5 (C-4’), 143.8 (C-3’), 134.26 (C-3), 121.8 (C-6’), 121.3 (C-1’), 116.0 (C-2’), 115.2 (C-5’),104.1 (C-10), 102.5 (C-1’’’),100.4 (C-1’’), 98.5 (C-6), 93.6 (C-8), 77.7 (C-5’’), 77.3 (C-3’’), 74.7 (C-2’’), 73.6 (C-4’’’), 70.8 (C-2’’’), 70.7 (C- 3’’’), 70.4 (C-4’’), 69.7 (C-5’’’), 66.9 (C-6’’), and 18.4 (C-6’’’).

3’,4’-dimethoxy quercetin 3-*O*-neohispredoside (**3**): Off-white amorphous powder, gives dark purple spot under long UV light λ _max_ 365 turned to orange with Naturstoff spray reagent. Negative ESI-MS: *m/z* 637.18 [M–H]^–^, 491.12 [M–H–rahmnose]^–^, 329.07 [M–H–rahmnose-glucose]^–^ = [aglycone–H]^–^.^1^ H NMR (300 MHz, DMSO-*d*_6_): *δ* = 7.64 (1 H, dd, *J* = 8.4, 1.2 Hz, H-6’), 7.52 (1 H, d, *J* = 1.2 Hz, H-2’), 6.82 (1 H, d, *J* = 8.4 Hz, H-5’), 6.43 (1 H, d, *J* = 2.1 H-8), 6.16 (1 H, d, *J* = 2.1 H-6), 5.64 (1 H, d, *J* = 7.8 Hz, H-1’’), 4.23 (1 H, d, *J* = 7.8 Hz, H-1’’’), 3.83(3 H, s, methoxy at C-4’), 3.81(3 H, s, methoxy at C-3’), 3.73–3.31 (remaining sugar protons), and 0.76 (3 H, d, *J* = 6, H-6’’’). ^13^C NMR (75 MHz, DMSO-*d*_6_): *δ* = 177.3 (C-4), 164.0 (C-7), 161.3 (C-5), 156.3 (C-2), 156.1 (C-9) 148.4 (C-4’), 144.9 (C-3’), 132.91 (C-3), 121.7 (C-6’), 121.2 (C-1’), 116.0 (C-2’), 115.2 (C-5’), 104.1 (C-10), 103.8 (C-1’’’), 100.6 (C-1’’) 98.4 (C-6), 93.6 (C-8), 77.5 (C-5’’), 77.3 (C-3’’), 74.7 (C-2’’), 73.5 (C-4’’’), 70.7 (C-2’’’), 70.7 (C-3’’’), 70.3 (C-4’’), 69.7 (C-5’’’), 66.2 (C-6’’), 56.9 ( methoxy at C-4’), 55.6 ( methoxy at C-3’), and 18.2 (C-6’’’).

Hedragenin 3-*O*-*β*-**D**-^4^C_1_-glucopyranoside (**4**): Off-white amorphous powder. Negative ESI-MS: *m/z* = 633.40 [M–H]^–^, 471.34 [M–H–glucose]^–^ = [aglycone -H]^–^. ^1^H NMR (500 MHz, pyridine-*d*_5_): *δ* = 6.16 (1H, brs, H-12), 5.06 (d, *J* = 7.5, H-1’), 4.27 (1H, m, H-3), 3.73 (1H, d, *J* = 10.5, H-23 a), 3.71(1H, d, *J* = 10.5, H-23 b), 3.26 (1H, dd, *J* = 13.5, 3.5, H-18), 1.16 (3 H, s, Me-27), 1.09 (3 H, s, Me-24), 1.07 (3 H, s, Me-26), ) 0.99 (3 H, s, Me-25), 0.92 (3 H, s, Me-30), and 0.89 (3 H, s, Me-29). ^13^C NMR (125 MHz, pyridine-*d*_5_): *δ* = 179.8 (C-28), 144.4 (C-13),122.2 (C-12), 101.3 (C-1’), 80.3 (C-3), 77.4 (C-5’), 76.3 (C-3’), 74.4 (C-2’), 70.4 (C-4’), 63.3 (C-23), 62.4 (C-6’), 48.0 (C-9), 47.4 (C-17), 46.8 (C-5), 46.0 (C-19), 43.2 (C-4), 41.9 (C-18), 41.8 (C-14), 39.3 (C-8), 38.7 (C-1), 36.6 (C-10), 33.9 (C-21), 32.8 (C-22), 32.5 (C-7), 32.8 (C-22), 30.5 (C-20), 27.8 (C-15), 25.7 (C-27, C-2), 23.6 (C-11,C-30), 23.2 (C-16), 17.6 (C-6), 17.3 (C-26),16.0 (C-25), and 13.8 (C-24)^50^.

Hedragenin 3-O-α-L-C-rahmnopyranoside (**5**): Off-white amorphous powder. Negative ESI-MS: *m/z* = 617.40 [M–H]^–^, 471.34 [M–H–rahmnose]^–^ = [aglycone -H]^–^. ^1^H NMR (500 MHz, pyridine-*d*_5_): *δ* = 6.27 (1H, brs, H-12), 5.46 (1H, brs, H-1’), 4.27 (1H, m, H-3), 3.74 (1H, d, *J* = 10.5, H-23 a), 3.69 (1H, d, *J* = 10.5, H-23 b), 3.29 (1H, dd, *J* = 13.5, 3.5, H-18), 1.22 (3 H, s, Me-27), 1.06 (3 H, s, Me-26), 1.02 (3 H, s, Me-24), 0.99 (3 H, s, Me-25), 0.93 (3 H, s, Me-30), and 0.92 (3 H, s, Me-29). ^13^C NMR (125 MHz, pyridine-*d*_5_): *δ* = 179.9 (C-28), 144.5 (C-13),122.3 (C-12), 101.5 (C-1’), 80.7 (C-3), 75.4 (C-4’), 72.2 (C-2’), 72.0 (C-3’), 69.0 (C-5’), 63.6 (C-23), 47.9 (C-9), 47.3 (C-17), 46.3 (C-5), 46.1 (C-19), 43.1 (C-4), 41.8 (C-14), 41.6 (C-18), 39.3 (C-8), 38.8 (C-1), 36.6 (C-10), 33.9 (C-21), 33.0 (C-29), 32.8 (C-22), 32.4 (C-7), 30.5 (C-20), 27.0 (C-15), 25.8 (C-27, C-2), 23.5 (C-11,C-30), 23.3 (C-16), 18.0 (C-6’), 17.8 (C-6), 17.1 (C-26),15.6 (C-25), and 13.5 (C-24)^50^.

Isoquercetien (**6**): Yellow amorphous powder, gives dark purple spot under long UV light λ _max_ 365, turned to orange with Naturstoff spray reagent. ^1^H NMR (300 MHz, DMSO-*d*_6_): *δ* = 7.66 (1H, d, *J* = 2.1 Hz, H-2’), 7.54 (1H, dd, *J* = 8.4, 2.1 Hz, H-6’), 6.88 (1H, d, *J* = 8.4 Hz, H-5’), 6.39 (1H, d, *J* = 1.5 H-8), 6.16 (1H, d, *J* = 1.5 H-6), 5.62 (1H, d, *J* = 7.8 Hz, H-1’’), and 3.47–3.61 (remaining sugar protons). ^13^C NMR (75 MHz, DMSO-*d*_6_): *δ* = 175.8 (C-4), 164.5 (C-7), 160.7 (C-5), 156.8 (C-2), 156.2 (C-9) 147.5 (C-4’), 145.1 (C-3’), 132.7 (C-3), 121.9 (C-6’), 119.9 (C-1’), 115.9 (C-2’), 115.1 (C-5’), 102.8 (C-10), 100.0 (C-1’’), 98.4 (C-6), 93.6 (C-8), 77.2 (C-5’’), 76.8 (C-3’’), 73.3 (C-2’’), 69.8 (C-4’’), and 60.7 (C-6’’).

*E*-caffeic acid 4-*O*-*β*-**D**-^4^C_1−_glucopyranoside (**7**): Off-white amorphous powder gives blue color under long UV light λ _max_ 365.^1^ H NMR (500 MHz, DMSO-*d*_6_): *δ* = 7.42 (1H, d, *J* = 15.5 Hz, H-7), 7.04 (1H, d, *J* = 2, H-2), 6.96 (1H, dd, *J* = 8.0, 2.0 Hz, H-6), 6.87 (1H, d, *J* = 8.0 Hz, H-5), 6.18 (1H, d, *J* = 15.5 Hz, H-8), 4.93 (1H, d, *J* = 7.5 Hz, H-1”), and 3.16–3.68 (remaining sugar protons). ^13^C NMR (125 MHz, DMSO-*d*_6_): *δ* = 168.5 (C-9), 147.4 (C-4), 145.7 (C-7), 145.3 (C-3), 128.5 (C-1), 121.6 (C-6), 115.9 (C-5), 115.1 (C-2), 114.9 (C-8), 100.8 (C-1’), 77.9 (C-5’), 77.5 (C-3’), 74.8 (C-2’), 70.7 (C-4’), and 60.9 (C-6’) S1

*E*-caffeic acid (**8**): yellowish white powder, gives blue color under long UV light λ _max_ 365. ^1^H NMR (400 MHz, DMSO-*d*_6_): *δ* = 7.57 (1H, d, *J* = 15.9 Hz, H-7), 7.05 (1H, d, *J* = 2.0, H-2), 6.75 (1H, dd, *J* = 8.0, 2.0 Hz, H-6), 6.787 (1H, d, *J* = 8.0 Hz, H-5), and 6.28 (1H, d, *J* = 15.5 Hz, H-8). ^13^C NMR (100 MHz, DMSO-*d*_6_): *δ* = 167.5 (C-9), 148.5 (C-4), 145.3 (C-7), 145.2 (C-3), 127.9 (C-1), 121.6 (C-6), 115.8 (C-5), 115.4 (C-2), and 114.6 (C-8).

### Antischistosomal activity

The antischistosomal potential of *C. madurensis* leaves extracts was investigated using the in vitro worm killing of *S. mansoni* (Table 2). The results (Table 2) revealed that four extracts and five fractions from crude aqueous methanol extract showed, antischistosomal potential in vitro. At the examined concentration tested (100 µg/mL), the active *C. madurensis* samples caused paralysis followed by death; where worms appeared longer, thinner followed by death. The antischistosomal activity seemed to be clearly evident with some extracts and fractions than others. Fraction V was the most effective revealing 95% worm killing followed by that of fractions VI and VII. The activity variation may be due to differences in chemical nature and quantity of active substances released using different solvents employed in the extraction process as well as to various elution systems polarity used in chromatographic fractionation^67^.

The antischistosomal activity of fraction V can be attributed to its flavonol content, particularly the acylated flavonol (*Crotalamad*oside A), which has been the major component in this fraction.

**Table 2:** *In vitro S. mansoni* worm killing using *C. madurensis* different extracts including, the crude methanol extract and its isolated fractions.

Extract/fraction	Elution systems	% Worm killing
CH_2_Cl_2_ extract (A)		5%
EtOAc extract (B)		50%
*n*-BuOH extract (C)		80%
Desalting crude MeOH 100% extract (D) and its fractions		
Fraction I	100% H_2_O	0%
Fraction II	5% MeOH	0%
Fraction III	5% MeOH	0%
Fraction IV	10% MeOH	0%
Fraction V	20% MeOH	95%
Fraction VI	40% MeOH	50%
Fraction VII	60% MeOH	40%
Fraction VIII	80% MeOH	20%
Fraction IX	100% MeOH	20%
Negative DMSO & Methanol		0%
PZQ		100%

Concentration tested = 100 µg/mL.

Regarding antiparasitic activities of flavones and flavonols, they were found to possess antimalarial^68,69^ and antischistosomal activities^68^. The flavonol quercetin is the main common moiety in the fraction V compounds, which was reported to have effective potential on protozoa such as malaria, trypanosome, toxoplasma and leshmenia^70,71^. In addition, it has effective potential on trematodes (*S. Mansoni* and *Fasciola hepatica*)^72–74^. Quercetin availed also as a result of the chromatographic isolation of *C. madurensis* has been known as a selective inhibitor of the *S. mansoni* NAD+ catabolizing ectoenzyme (SmNACE) located on the adult parasite tegument outer surface potentially involved in the parasite’s survival^72^ through cleavage of Nicotinamide Mono Nucleotide “NMN” to generate the vital nicotinamide (vitamin B3) for convenient uptake by the worms^75^.

Acylation of flavonol by cinnamic acid derivatives as in the case of (*Crotalamadoside *A) (**1**) may improve its parasitic activity because acylated flavonoids were also reported to have antiparasitic activity involving antimalarial^76^ and antischistosomal activities^73^. In addition, caffeic acid itself exhibits antiparasitic activity by inhibiting the growth and viability of various protozoa, including *Leishmania*, *Trypanosoma*^77^ and malaria^78^ as well as schistosoma^79^. Furthermore, the association of sulfated group to the external glucose unit of compound **1** may be playing a role in the enhancement of the antischistosomal activity of fraction V, as sulfated sugars were reported to prevent parasite’s adhesion to host cells, disrupt its life cycle, and modulate the host’s immune responses to eliminate the infection. The sulfate negative charge allows interaction with the proteins on the parasite surface and the receptors of host cell effectively blocking invasion and serving as a foundation for novel drug^80^. The demonstrated antischistosomal activity of fraction VI (Table 2) can be attributed to triterpenoidal saponin compounds synergistic effects, which is in harmony with our previous findings where antischistosomicidal activity was recorded in vivo for the crude saponin isolated from *C. madurensis* aerial parts *n*-butanol extract^3^.

## Conclusion

This research demonstrated the potential anti-schistosomal activity of various extracts of *C. madurensis* using the in vitro schistosome worm killing where most of antischistosomal drug discovery rely on, the assay have the advantage of allowing for multiple dosing systems over a short duration. The results revealed that the most active extract was the fat- and salt-free methanol aqueous extract (70%), which was chromatographically fractionated to test the effectiveness of its different fractions in vitro against adult schistosomes. Data revealed also, the identification of certain flavonoids during the phytochemical analyses that are likely responsible for some of the demonstrated antischistosomal activity, expanding the horizons of antiparasitic research. Findings stimulate further researches to clarify the medical impact of the most effective anti-parasitic compounds of *Crotalaria* species in the treatment of various parasitic illnesses.

Given that *C*. *madurensis* revealed effective antischistosomal activity; further researches should be conducted to unveil other significant antiparasitic activities of this genus. Meanwhile, since compound (*Crotalamadoside* A) (**1**) constitute the main component in the fraction “V” that showed potent schistosomicidal activity; it is assumed that the activity recorded can be attributed to this compound. Furthermore, the three compounds share the same aglycone (quercetin), and the activity of fraction"V”, may result from a positive synergistic effect between them. Extensive biological assays preceding primary clinical studies should be targeted to confirm the effectiveness of these acylated flavonol, with the aim of facilitating the application of these natural compounds in the drugs industries for the treatment of parasites and other diseases.

## Future prospects

Based on the promising initial results obtained so far, the research team will continue studying biochemical monitoring through activity assays to obtain the in vitro LC_90_ for the active fractions, aiming to evaluate the compounds responsible for the in vivo schistosomicidal activities of this medicinal plant under investigation.

## Supplementary Information

Below is the link to the electronic supplementary material.

Supplementary Material 1

## Data Availability

All data generated or analyzed during this study are included in this published article.

## Abbreviations

TLC: Thin layer chromatography
PC: Paper chromatography
2D-PC: Two dimensional paper chromatography
ESI-MS: Electrospray spray ionization
¹H NMR: Proton nuclear magnetic resonance
¹³C NMR: Carbon-13 nuclear magnetic resonance
¹H–¹H COSY: Proton–proton correlation spectroscopy
HSQC: Heteronuclear single quantum coherence
HMBC: Heteronuclear multiple bond correlation
CC: Column chromatography
TLC: Thin layer chromatography
SBSC: Schistosome biological supply center
Co-TLC: Co-thin layer chromatography
Co-PC: Co-paper chromatography
R_f_: Retention factor
m/z: Mass-to-charge ratio
J: Coupling constant
δ: Chemical shift
ppm: Parts per million
MHz: Megahertz
hrs: Hours
BuOH: Butanol
B. alexandrina: Biomphalariae alexandrina
TBRI: Theodor bilharz research institute
PZQ: Praziquantel

## References

[1] Nwude, N. & Ibrahim, M. A. Plants used in traditional veterinary medical practice in Nigeria. *J. Vet. Pharmacol. Ther.* **3**, 261–273. 10.1111/j.1365-2885.1980.tb00491.x (1980).

[2] Pandey, A. & Nayar, E. R. Some observations on systematics of *Crotalaria* species. *J. Plant. Genet. Resour.* **7**, 133–145 (1994).

[3] Ibrahim, M. T., Mohamed, M. A., Mohamed, H. S. & Mahmoud, M. R. Phytochemical and biological studies on *Crotalaria madurensis* (Family Fabaceae). *Int. J. Pharmacogn. Phytochem. Res.* **9**, 355–363 (2017).

[4] Mohammed, H. S., Abu El Wafa, S. A., Ibrahim, M. H. & Fathy, R. M. Seif- Eldein, N A. *Crotalaria madurensis* flavonol glycosides’ antibacterial activity against *Staphylococcus aureus*. *AMB Express*. **14**, 3–19. 10.1186/s13568-024-01776-3 (2024). 39495369 10.1186/s13568-024-01776-3 PMC11535145

[5] Hala, S., Mohamed, S., Magada, T., Batran, S. E. & Omayma, D. Phytochemical and pharmacological studies of *Crotalaria madurensis* leaves. *Planta Med.* **74**, PA344. 10.1055/s-0028-1084341 (2008).

[6] Andriamampianina, H. L. et al. B. Antimicrobial guanidine alkaloids from the leaves of *Crotalaria bernieri* Baill (Fabaceae). *GSC Biol. Pharm. Sci.* **29**, 27–37. 10.30574/gscbps.2024.29.3.0456 (2024).

[7] Baba, N. D. & Njoku, C. O. Phytochemistry of the methanolic leaf extract of *Crotalaria lachnosema* Stapf. (Fabaceae) and acute exposure to the extract-induced clinico-pathological changes in male wistar rats. *ARC J. Anim. Vet. Sci.* **3**, 24–28. 10.20431/2455-2518.0303004 (2017).

[8] Tang, X. et al. Simultaneous extraction and separation of flavonoids and alkaloids from *Crotalaria sessiliflora* L. by microwave-assisted cloud-point extraction. *Sep. Purif Technol*. **175**, 266–273. 10.1016/j.seppur.2016.11.038 (2017).

[9] Hu, X. R., Chou, G. X. & Zhang, C. G. Flavonoids, alkaloids from the seeds of *Crotalaria pallida* and their cytotoxicity and anti-inflammatory activities. *Phytochemistry* **143**, 64–71. 10.1016/j.phytochem.2017.07.010 (2017). 28777979 10.1016/j.phytochem.2017.07.010

[10] Scupinari, T. et al. *Crotalaria spectabilis* as a source of pyrrolizidine alkaloids and phenolic compounds: HPLC-MS/MS dereplication and monocrotaline quantification of seed and leaf extracts. *Phytochem Anal.* **31**, 747–755. 10.1002/pca.2938 (2020). 32428987 10.1002/pca.2938

[11] Recha, C., Ribeirob, L. P., Bentoc, J. M. S., Potta, C. A. & Nardia, C. Monocrotaline presence in the *Crotalaria* (Fabaceae) plant genus and its influence on arthropods in agroecosystems. *Braz J. Biol.* **84**, e256916. 10.1590/1519-6984.256916 (2024). 10.1590/1519-6984.256916 35043839

[12] Zhang, W. et al. Ultra-performance liquid chromatography hyphenated with quadrupole-orbitrap mass spectrometry for simultaneous determination of necine-core-structure pyrrolizidine alkaloids in *Crotalaria sessiliflora* L. without all corresponding standards. *Phytochem Anal.* **28**, 365–373. 10.1002/pca.2683 (2017). 28332747 10.1002/pca.2683

[13] Anwar, S. et al. A comprehensive phytochemical, biological, and toxicological studies of roots and aerial parts of *Crotalaria burhia* Buch.-Ham: An important medicinal plant. *Front. Plant. Sci.* **13**, 988352. 10.3389/fpls.2022.988352 (2022). 36212347 10.3389/fpls.2022.988352 PMC9533709

[14] Anza, M. & Gelaw, H. Preliminary phytochemical investigation of pod and seed extract of *Crotalaria incana* L. subsp. *Purpurscens*. *J. Coast Life Med.* **3**, 555–556. 10.12980/JCLM.3.2015J5-31 (2015).

[15] Panda, S. K., Das, D. & Treaty, N. K. Screening of antidiabetic activity of leaf extracts of *Crotalaria pallida* in alloxan induced diabetic rats. *Der Pharmacia Sinica*. **6**, 41–44 (2015).

[16] Mazumder, T. et al. M.S.U. Phenolic compounds and extracts from *Crotalaria calycina* Schrank potentially alleviate pain and inflammation through inhibition of cyclooxygenase-2: An *in vivo* and molecular dynamics studies. *Heliyon*. 8, e12368 10.1016/j.heliyon.2022.e12368 (2022). 10.1016/j.heliyon.2022.e12368 PMC9800535 36590510

[17] Narender, T., Tanvir, K., Rao, M. S., Srivastava, K. & Puri, S. K. Prenylated chalcones isolated from *Crotalaria* genus inhibits *in vitro* growth of the human malaria parasite *Plasmodium falciparum*. *Bioorg. Med. Chem. Lett.* **15**, 2453–2455. 10.1016/j.bmcl.2005.03.081 (2005). 15929201 10.1016/j.bmcl.2005.03.081

[18] Namratha, V. Phytochemical investigation of *Crotalaria* species-isolation of a new dihydro chalcone from *Crotalaria ramosissima*, Current Aspects in Pharmaceutical Research and Development. *Book. Publisher Int. (apart Sci. domain international)*. **2**, 169–182. 10.9734/bpi/caprd/v2/13153D (2021).

[19] Umashankar, T., Govindappa, M., Yarappa, R. L., Rai, P., Ryavalad, C. & S. & Isolation and characterization of coumarin isolated from endophyte, *Alternaria* species-1 of *Crotalaria pallida* and its apoptotic action on HeLa cancer cell line. *Metabolomics* **5**, 158. 10.4172/2153-0769.1000158 (2015).

[20] Paulpriya, K., Tresina, P. S. & Mohan, V. R. Isolation, purification and characterization of 2,2-dimethylchromene 7-methoxy-6-*O*-β-glucopyranoside (chromene derivatives) from *Crotalaria longipes* Wight & Arn. *Chem. Sci. Rev. Lett.* **4**, 1269–1274 (2015).

[21] Fadzil, S. R., Yap, A. C. & Choo, Y. M. A new cyclopentylidene and other chemical constituents from Malaysian *Crotalaria pallida*. *Sains Malays*. **46**, 1581–1586. 10.17576/jsm-2017-4609-29 (2017).

[22] Kang, D. G., Lee, Y. S., Kim, H. J., Lee, Y. M. & Lee, H. S. Angiotensin converting enzyme inhibitory phenylpropanoid glycosides from *Clerodendron trichotomum*. *J. Ethnopharmacol.* **89** (03), 151–154. 10.1016/s0378-8741 (2003). 14522447 10.1016/s0378-8741(03)00274-5

[23] Fan, C. M., Chou, G. X. & Zhu, E. Y. Chemical constituents from *Crotalaria sessiliflora *L.* Yao Xue Xue Bao*. **51**, 775–779 (2016). 29877686

[24] Zou, Y. H., Liu, X., Liu, Y. N., Tang, G. H. & Yin A novel heterodimer from *Crotalaria ferruginea*. *Nat. Prod. Commun.* **11**, 793–794 (2016). 27534118

[25] Kodiralieva, F. K., Shashkov, A. S. & Rakhmanberdyeva, R. K. Structure of galactomannan from seeds of *Crotalaria alata*. *Chem. Nat. Compd.* **51**, 405–408. 10.1007/s10600-015-1303-y (2015).

[26] Soni, B. Preliminary phytochemical screening and antimicrobial activity of methanol extract of *Crotalaria burhia*. *Pharma Tutor.* **2**, 115–118 (2014).

[27] Sikta, S. A. Extraction and quantitation of total peptides and proteins from the whole plant *Crotalaria pallida* and determination of antimicrobial activity of the isolated peptides and proteins. A project submitted. In *partial fulfillment requirements degree Bachelor Pharm. (Hons)*, (2017).

[28] Chouhan, H. S., Sahu, A. N. & Singh, S. K. Fatty acid composition, antioxidant, anti-inflammatory and antibacterial activities of seed oil from *Crotalaria juncea* Linn. *J. Med. Plants Res.* **5**, 984–991 (2011).

[29] Alemu, M. A., Mekonnen, H. G. & Annisa, M. E. Phytochemical analysis and antibacterial activity on seed and pod extracts of *Crotalaria incana*. *J. Pharm. Pharmacogn Res.* **3**, 100–108. (2015).

[30] Kumar, G. G., Gali, V. & Dwivedi, S. C. Phytochemical investigation of *Crotalaria burhia *Hamilt. *Int. J. Pharm. Sci. Res.* **2**, 1721–1724 (2011).

[31] Ravi Shankar, K. & Gnaneswari, K. Thirumala Devi, S. Exploring the biological activities of *Crotalaria juncea* whole plant for its therapeutic potential. *Ind. J. Nat. Sci.* **16**, 95541–95548. 10.5114/bta.2023.132772 (2025).

[32] Mazumder, T. et al. M. S. U. Phenolic compounds and extracts from *Crotalaria calycina* Schrank potentially alleviate pain and inflammation through inhibition of cyclooxygenase-2: An *in vivo* and molecular dynamics studies. *Heliyon* **8**, e12368. 10.1016/j.heliyon.2022.e12368 (2022). 36590510 10.1016/j.heliyon.2022.e12368 PMC9800535

[33] Honório Júnior, J. E. et al. Pharmacological activity of monocrotalina isolated from plants of the genus *Crotalaria*. *Rev. Bars Farmacogn*. **20**, 453–458. 10.1590/S0102-695X2010000300025 (2010).

[34] Rocha, L. G. et al. E. F. Evaluation of the leishmanicid action of ethanolic extracts of *Crotalaria retusa* L. (Fabaceae). *Rev. Bars Farmacogn*. **19** (1A), 51–56. 10.1590/S0102-695X2009000100012 (2009).

[35] Ramya, L. B., Mohan, L. S. & Sharavana, K. A. Evaluation of antidiarrheal activity of methanolic extract of *Crotalaria juncea* Linn in albino Wistar rats. *Int. J. Preclin Pharm. Res.* **2**, 66–70 (2011).

[36] Adenusi, A. A. & Odaibo, A. B. A laboratory assessment of the potential molluscicidal activity of some Nigerian plant species used as anthelmintics. *Afr. J. Aquat. Sci.* **35**, 251–258. 10.2989/16085914.2010.545506 (2010).

[37] Alam, M. H. et al. Assessment of antioxidant, antibacterial and anthelmintic activities of ethanol extract of leaves of *Crotalaria pallida* (Aiton). *Int. J. Pharmacogn*. **1**, 438–444. 10.13040/IJPSR.0975-8232.IJP.1(7) (2014).

[38] Mahasawat, P., oukaew & Prasertsan, S. Exploring the potential of *Crotalaria juncea* flower extracts as a source of antioxidants, antimicrobials, and cytoprotective agents for biomedical applications. *BioTechnologia* **104**, 359–370 (2023). 38213478 10.5114/bta.2023.132772 PMC10777724

[39] Lekharani, C., Yanadaiah, J. P., Ravindra Reddy, K., Lakshman Kumar, D. & Venkatasubbaiah, M. Hepatoprotictive activity of aqueous ethanolic extract of aerial parts of *Crotalaria verrucosa* Linn paracetamol-induced hepatotoxicity in rats. *J. Pharm. Biol. Sci.* **1**, 50–55 (2013).

[40] Paulpriya, K., Tresina, P. S. & Mohan, V. R. Hepatoprotective effect of *Crotalaria longipes* Wight & Arn, ethanol extract in CCl_4_ induced hepatotoxicity in wistar rats. *Int. J. Toxicol. Pharmacol. Res.* **8**, 45–52 (2016).

[41] Mohamed, M. A. Spirostanol saponin from *Asparagus sprengeri* and their molluscicidal activity. *Nat. Prod. Commun.* **2**, 731–736. 10.1177/1934578X0700200705 (2007).

[42] Mohamed, M. A., Hayen, H. & Mahmoud, M. R. Evaluation of antinociceptive and anti-infmmatiory activates of a new triterpene saponin from *Bauhinia vatriegata* leaves. *Z. Naturforsc*. **64C**, 798–808. 10.1515/znc-2009-11-1208 (2009). 10.1515/znc-2009-11-1208 20158149

[43] William, S. et al. Potential antischistosomal activities of some Egyptian native plants using *Schistosoma mansoni* worm killing assay. *Glob J. Pharmacol.* **8**, 237–244. 10.5829/idosi.gjp.2014.8.2.8379 (2014).

[44] Seif el-Din, S. H. et al. Potential effect of the medicinal plants* Calotropis procera*, *Ficus**elastica* and *Zingiber officinale* against *Schistosoma mansoni* in mice. *Pharm. Biol.* **52**, 144–150. 10.3109/13880209.2013.818041 (2014). 24047470 10.3109/13880209.2013.818041

[45] Kitajima, J., Shindo, M. & Tanaka, Y. Two new triterpenoid sulfates from the leaves of *Schefflera octophylla*. *Chem. Pharm. Bull.* **38**, 714–716. 10.1248/cpb.38.714 (1990).

[46] Liang, Y. S., Bruce, Y. S. & Boy, D. A. Laboratory cultivation of schistosome vector snails and maintenance of schistosome life cycles. *Proc. First Sino-Am. Symp*. 34–48 (1987).

[47] Agrawal, P. K. & Bansal, M. C. *Carbon-13 NMR of Flavonoids* Vol. 39, 283 (Eds) Elsevier, 1989).

[48] Harborne, J. B., Mabry, T. J. & Mabry, H. The Flavonoids: Advances in Research, Chap. 2, Chapman and Hall London (1982).

[49] Metri, S., Singh, R. P. & Shah, M. Isolation and identification of isoquercetin –A flavonoid from *Bryonia lacinosa*. *Int. J. Adv. Res.* **7**, 780–786. 10.21474/IJAR01/9889 (2019).

[50] Azimova, S. S. Natural compounds triterpene glycosides, Plant sources structure and properties, Springer New York Heidelberg Dordrecht London, (2013).

[51] Woo, K. W. et al. Phytochemical constituents from the rhizomes of *Osmunda japonica* Thunb and their anti-oxidant activity. *Nat. Prod. Sci.* **23**, 217–221. 10.20307/nps.2017.23.3.217 (2017).

[52] Abdul-Jalil, T. Z., Ibrahim, R. M. & Nassir, Z. S. Extraction, isolation and structure elucidation of two phenolic acids from aerial parts of Celery and Coriander. *Biomed. Pharmacol. J.* **16**, 2315–2332. 10.13005/bpj/2807 (2023).

[53] Tomas-Barberan, F. A., Gil, M. I., Ferrenres, F. & Tomas-Lorente, F. Flavonoid p-coumaroyl and 8-hydroxy flavone allosyl glucosides in some Labiatae. *Phytochemistry* **31**, 3097–3102. (1992).

[54] Duong, T. H. et al. Sulfonic acid-containing flavonoids from the roots of *Phyllanthus acidus*. *J. Nat. Prod.* **28**, 2026–2031. 10.1021/acs.jnatprod.8b00322 (2018). 10.1021/acs.jnatprod.8b00322 30207470

[55] Woz´nicka, E. New sulfonic derivatives of quercetin as complexing reagents: synthesis, spectral, and thermal characterization. *J. Therm. Anal. Calorim.* **120**, 351–361. 10.1007/s10973-014-3677-7 (2015).

[56] Xi, Z. X. et al. Anti-complementary activity of flavonoids from *Gnaphalium affine* D. Don. *Food Chem.* **130**, 165–170. 10.1016/j.foodchem.2011.07.025 (2012).

[57] Mohamed, M. A., Ahmed, W. S., El-Said, M. M. & Hayen, H. New acylated flavonol diglycosides of *Cynanchum acutum*. *Nat. Prod. Commun.* **3**, 193–198. 10.1177/1934578X0800300 (2008).

[58] Mohamed, M. A., Hamed, M. H., Ahmed, W. S. & Abdou, A. M. Antioxidant and cytotoxic flavonols from *Calotropis procera*. *Z. Naturforsch*. **66 c**, 547–554. 10.1515/znc-2011-11-1203 (2011). 10.1515/znc-2011-11-1203 22351979

[59] Essa, A. F. et al. W. Natural acylated flavonoids: their chemistry and biological merits in context to molecular docking studies. *Phytochem Rev.* **22**, 1469–1508. 10.1007/s11101-022-09840-1 (2023).

[60] Mohamed, M. A., Hamed, H. M., Abdou, A. M., Ahmed, W. S. & Saad, A. M. Antioxidant and cytotoxic constituents from *Wisteria sinensis Molecules*. **16**, 4020–4030. 10.3390/molecules16054020 (2011).

[61] Qiu, M. F., Zhu, Y. D., Huang, W. H., Qi, Y. B. & Guo, L. L. Two new acylated flavonol glycosides from the seeds of *Lepidium sativum*. *Molecules* **19**, 11341–11349. 10.3390/molecules190811341 (2014). 25090122 10.3390/molecules190811341 PMC6271768

[62] Kuiate, T. T. et al. Marie-Aleth, L. D. Acylated triterpene saponins from *Atroxima liberica* Stapf. *Helv. Chim. Acta*. **94**, 2066–2076. 10.1055/s-0030-1264680 (2011).

[63] Sato, R., Nishidono, Y. & Tanaka, K. Comprehensive Analysis of Sulfated Flavonoids in *Eclipta prostrata* for Quality evaluation. *Molecules* **29**, 4888. 10.3390/molecules29204888 (2024). 39459257 10.3390/molecules29204888 PMC11509997

[64] Sweeny, J. G., Wilkinson, M. M. & Iacobucci, G. A. Effect of flavonoid sulfonates on the photobleaching of anthocyanins in acid solution. *J. Agric. Food Chem.* **29**, 563–567. 10.1021/jf00105a033 (1981).

[65] Santhanam, M., Hautala, R. R., Sweeny, J. G. & Iacobucci, G. A. The influence of flavonoid sulfonates on the fluorence and photochemistry of flavylium cations. *Photochem. Photobiol*. **38**, 477–480 (1983).

[66] Huang, Y. L., Chen, C. C., Hsu, F. L., Chen, C. F. & Tannins Flavonol Sulfonates, and a Norlignan from *Phyllanthus virgatus*. *J. Nat. Prod.* **61**, 1194–1197. 10.1021/np970336v (1998). 9784150 10.1021/np970336v

[67] Mansour, S. et al. Botanical biocides, Impact of some plant extracts on *Biomphalaria alexandrina* snails and on *Schistosoma mansoni* miracidia and cercariae. *Egypt. J. Sch. Infect. Dis.* **24**, 81–99 (2002).

[68] Wangchuk, P. et al. & Compounds derived from the Bhutanese Daisy, *Ajania nubigena*, demonstrate dual anthelmintic activity against *Schistosoma mansoni* and *Trichuris muris*. *PLos. Negl. Trop. Di.s*. 10, e0004908 (2016). 10.1371/journal.pntd.0004908 10.1371/journal.pntd.0004908 PMC4973903 27490394

[69] Kaur, K., Jain, M., Kaur, T. & Jain, R. Antimalarials from nature. *Bioorg. Med. Chem.* **17**, 3229–3256. 10.1016/j.bmc.2009.02.050 (2009). 19299148 10.1016/j.bmc.2009.02.050

[70] Gupta, A. K., Saxena, S. & Saxena, M. Integrated ligand and structure based studies of flavonoids as fatty acid biosynthesis inhibitors of *Plasmodium falciparum*. *Bioorg. Med. Chem. Lett.* **20**, 4779–4781. 10.1016/j.bmcl.2010.06.120 (2010). 20637612 10.1016/j.bmcl.2010.06.120

[71] Abugri, D. A. et al. Quercetin inhibits *Toxoplasma gondii* tachyzoite proliferation and acts synergically with azithromycin. *Parasit. Vectors*. **16**, 1–10. 10.1186/s13071-023-05849-3 (2023). 37537675 10.1186/s13071-023-05849-3 PMC10401810

[72] Kuhn, I. et al. Identification by high-throughput screening of inhibitors of *Schistosoma mansoni* NAD (+) catabolizing enzyme. *Bioorg. Med. Chem.* **18**, 7900–7910. 10.1016/j.bmc.2010.09.041 (2010). 20951593 10.1016/j.bmc.2010.09.041

[73] Braguine, C. G. et al. P. M. Schistosomicidal evaluation of flavonoids from two species of *Styrax* against *Schistosoma mansoni* adult worms. *Pharm. Biol.* **50**, 925–929. 10.3109/13880209.2011.649857 (2012). 22480261 10.3109/13880209.2011.649857

[74] pereir, C. A. J. et al. Anti-helmintic activity of *Momordica charantia* L. against *Fasciola hepatica* eggs after twelve days of incubation *in vitro*. *Vet. Parasitol.* **228**, 160–166. 10.1016/j.vetpar.2016.08.025 (2016). 27692319 10.1016/j.vetpar.2016.08.025

[75] Nation, C. S., Da’Dara, A. A. & Skelly, P. J. NAD-catabolizing ectoenzymes of *Schistosoma mansoni*. *Biochem. J.* **479**, 1165–1180. 10.1042/BCJ20210784 (2022). 35593185 10.1042/BCJ20210784

[76] Cai, S. et al. Identification of compounds with efficacy against Malaria parasites from common North American plants. *J. Nat. Prod.* **79**, 490–498. 10.1021/acs.jnatprod.5b00874 (2016). 26722868 10.1021/acs.jnatprod.5b00874 PMC5558429

[77] Bortoleti, B. T. D. S. et al. Caffeic acid has antipromastigote activity by apoptosis-like process; and anti-amastigote by TNF-α/ROS/NO production and decreased of iron availability. *Phytomed* **57**, 262–270. 10.1016/j.phymed.2018.12.035 (2019). 10.1016/j.phymed.2018.12.035 30802712

[78] Alson, S. G. et al. *In-vitro* and *in-vivo* antimalarial activity of caffeic acid and some of its derivatives. *J. Pharm. Pharmacol*. 70, 1349–1356 10.1111/jphp.12982 (2018). 10.1111/jphp.12982 30033538

[79] Da Silva Filho, A. A. et al. *vitro* antischistosomal activities of phenylpropanoids and lignans against *Schistosoma mansoni* adult worms. *Planta Med.* **75**, PD21. 10.1055/s-0029-1234500 (2009).

[80] Besednova, N. N. et al. Antiparasitic effects of sulfated polysaccharides from marine Hydrobionts. *Mar. Drugs*. **19**, 637. 10.3390/md19110637 (2021). 34822508 10.3390/md19110637 PMC8624348

