# Supplementary material for: Chemical profile and in vitro antischistosomal activity of Crotalaria madurensis different extracts
Source: Sci Rep. 2026 Jul 10;16:21615. doi: 10.1038/s41598-026-60749-7 (PMC13354784; doi:10.1038/s41598-026-60749-7)
Supplement: Supplementary file 1 — Supplementary Material 1 [file 41598_2026_60749_MOESM1_ESM.docx]

**Chemical profile and *in vitro* antischistosomal activity of *Crotalaria madurensis* different extracts**

Mona A. Mohamed^a,*^, Samia William^b^, Mosad A. Ghareeb^a,*^, Sanaa S. Botros^c^

^a^Medicinal Chemistry Department, Theodor Bilharz Research Institute, Kornaish El-Nile, Warrak El-Hadar, Imbaba (P.O. 30), Giza 12411, Egypt

^b^Parasitology Department, Theodor Bilharz Research Institute, Kornaish El-Nile, Warrak El-Hadar, Imbaba (P.O. 30), Giza 12411, Egypt

^c^Pharmacology Department, Theodor Bilharz Research Institute, Kornaish El-Nile, Warrak El-Hadar, Imbaba (P.O. 30), Giza 12411, Egypt

***Corresponding authors:**

Prof. Mona A. Mohamed; E-mail: [tbi20042003@hotmail.com](mailto:tbi20042003@hotmail.com)

Prof. Mosad A. Ghareeb; E-mail: [m.ghareeb@tbri.gov.eg](mailto:m.ghareeb@tbri.gov.eg); mosad.ghareeb@tbri.sci.eg

ORCID: <https://orcid.org/0000-0002-8398-1937>

**Abstract**

Chromatographic isolation of *Crotalaria madurensis* Wight & Arn leaves aqueous methanol extract availed a new sulfonic acid acylated flavonol glycosides named quercetin 8-potassium sulfonate 3-*O*-[2-*O*-sulfonyl]-*β*-D-^1^C_4_-glucopyranosyl-(1''''→3''')-4-*O*-[*E*- caffeoyl]-*β*-D-^4^C_1_-glucopyranosyl-(1'''→2'')-3-*O*-[*E*-caffeoyl]-*β*-D-^4^C_1_-glucopyranoside (*Crotalamad*oside A) (**1**), along with three known flavonoids identified as quercetin 7-*O*-neohispredoside (**2**), 3',4'-dimethoxy quercetin 3-*O*-neohispredoside (**3**) and isoquercetin (**6**) besides two triterpene saponin compounds named as hedragenin 3-*O*-*β*-D-^4^C_1_-glucopyranoside (**4**) and hedragenin 3-*O*-*α*-*L*-^1^C_4-_rhamnopyranoside (**5**). This was in addition, to two cinnamic acid derivatives named as *E*-caffeic acid 4-*O*-*β*-D-^4^C_1-_glucopranoside (**7**) and *E*-caffeic acid (**8**). Identification of the isolated compounds relied on their chromatographic properties and spectral data (UV, ESI-MS, ^1^H NMR, ^13^C NMR, ^1^H-^1^H COSY, HSQC, and HMBC). The antischistosomal activity of the ***C.*** *madurensis* different extracts were assessed *in vitro* using schistosome worm killing, findings revealed that the aqueous methanol extract was the most effective extract, chromatographic fractionation of this extract revealed significant antischistosomal activity for fractions number V and VI rich in flavonoids and triterpene saponins, respectively.

**Keywords:** *Crotalaria madurensis,* Antischistosomal***,*** Triterpenes***,*** Quercetin, Cinnamic acid derivatives, Good health and well-being

**Supplementary data for compound 1**

**
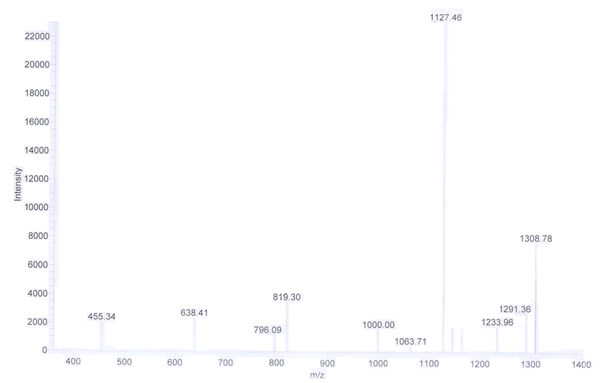
**

**Fig. S1.** Negative ESI-MS spectrum of Compound 1.


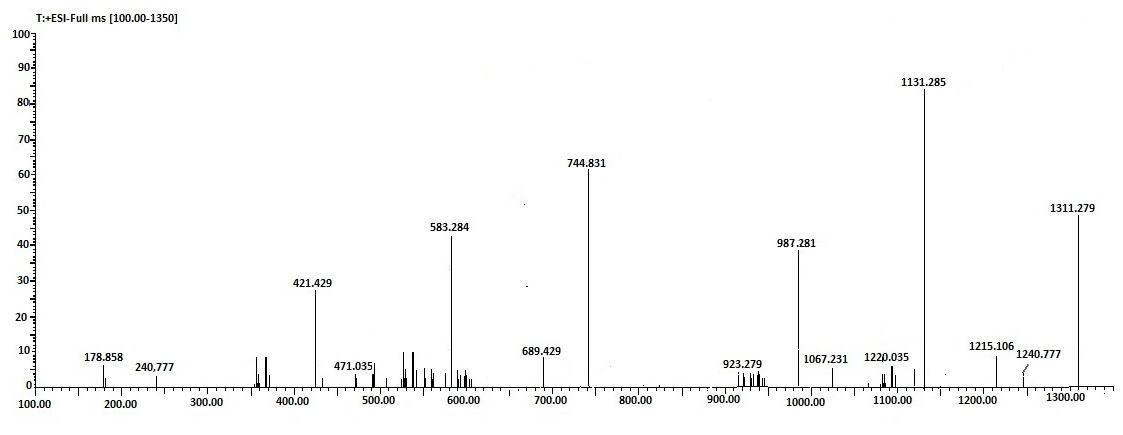


**Fig. S2.** Positive ESI-MS spectrum of Compound 1.

#
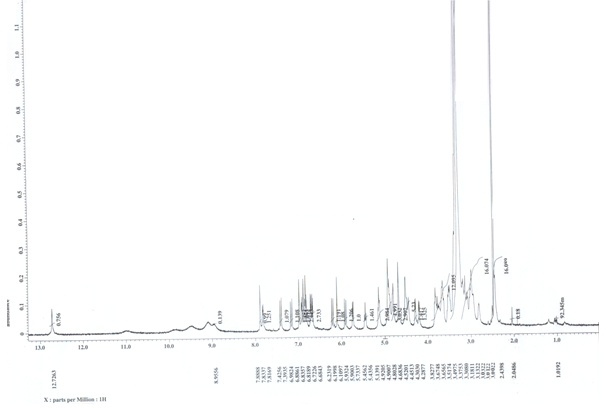


**Fig. S3**. Total ^1^H NMR spectrum of Compound 1.


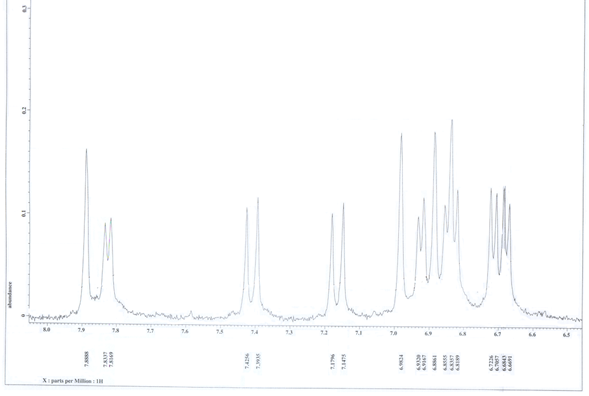


**Fig. S4**. ^1^H NMR spectrum of Compound 1 (part 1).


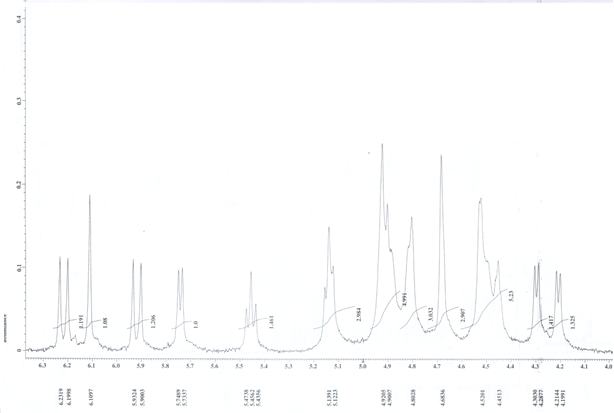


**Fig. S5**. ^1^H NMR spectrum of Compound 1 (part II).


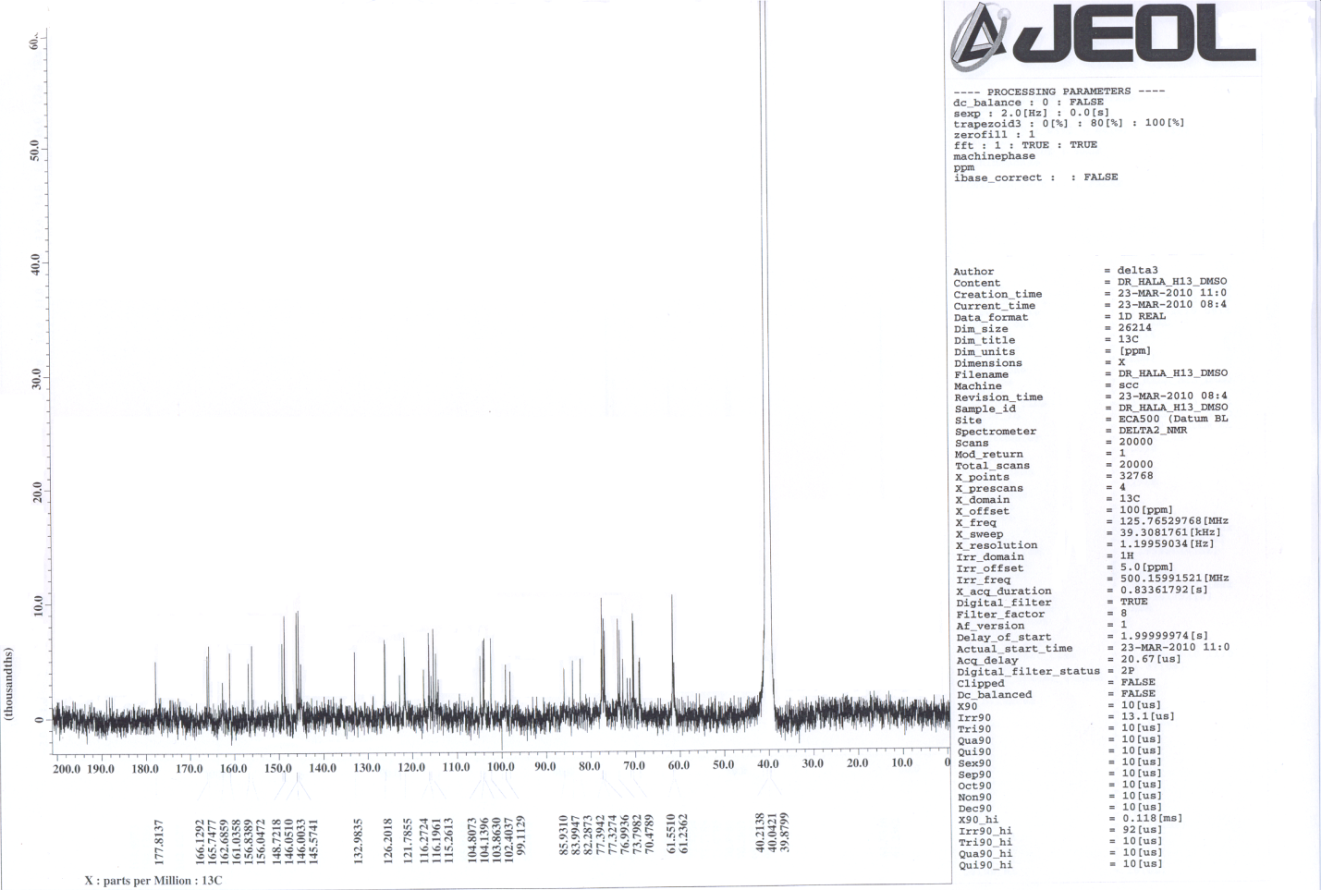


**Fig. S6**. Total ^13^C NMR spectrum of Compound 1.


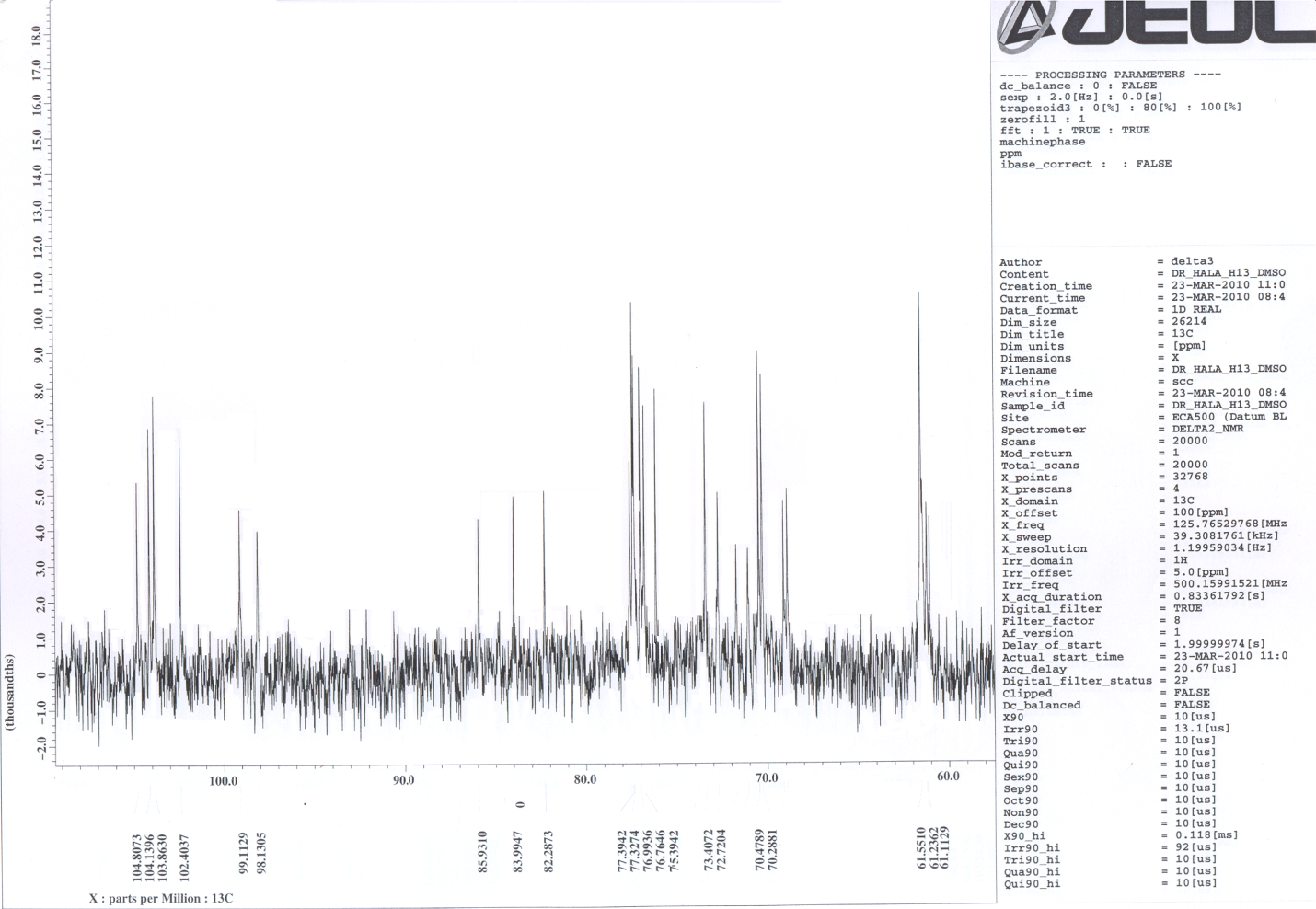


**Fig. S7**. ^13^C NMR of spectrum Compound 1(Part 1).


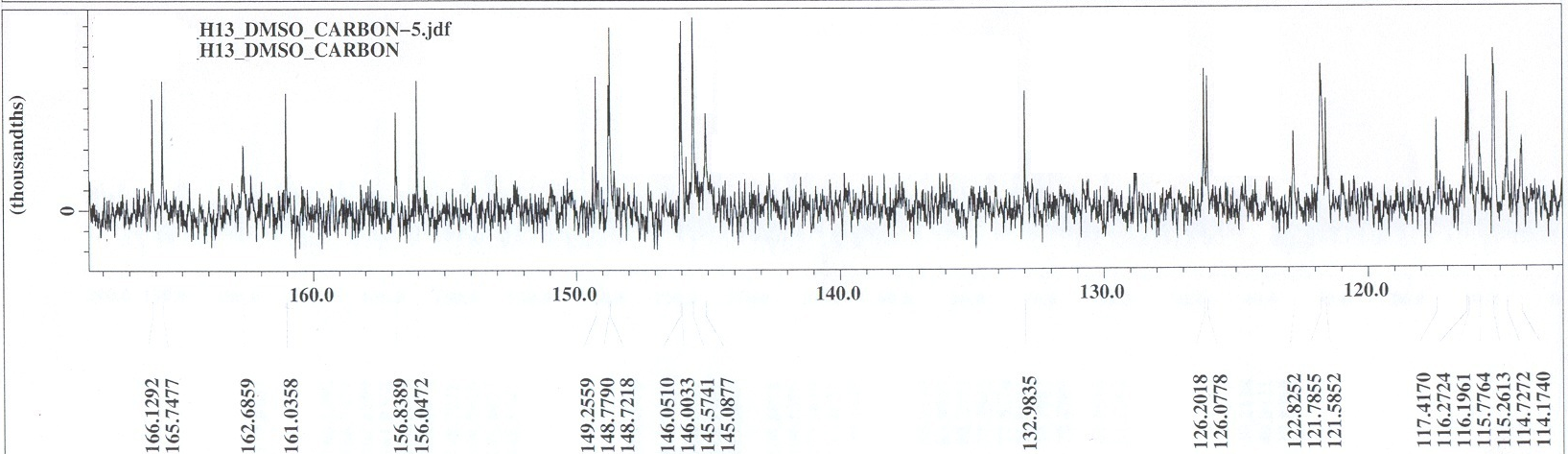


**Fig. S8**. ^13^C NMR spectrum of Compound 1(Part II).

**
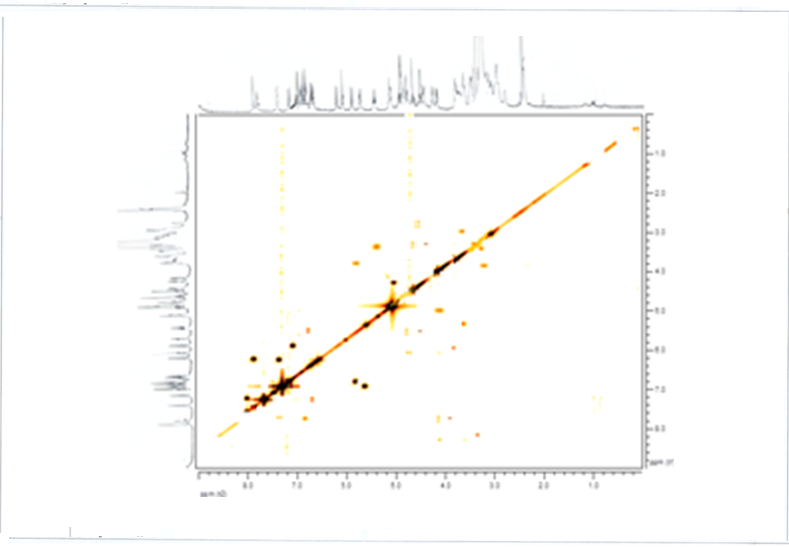
**

**Fig. S9**. ^1^H-^1^H-COSY spectrum of Compound 1.


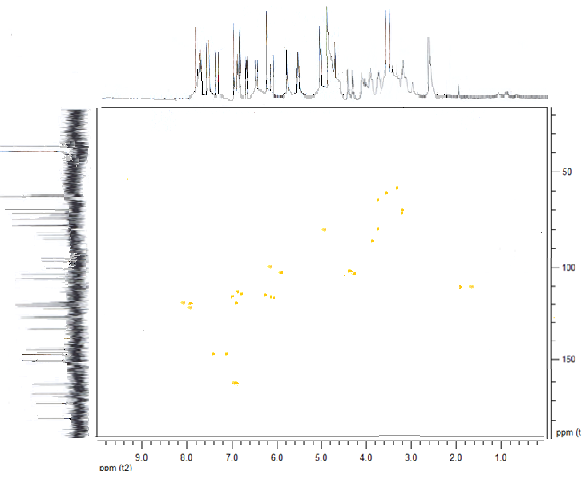


**Fig. S10**. HMQC spectrum of Compound 1.


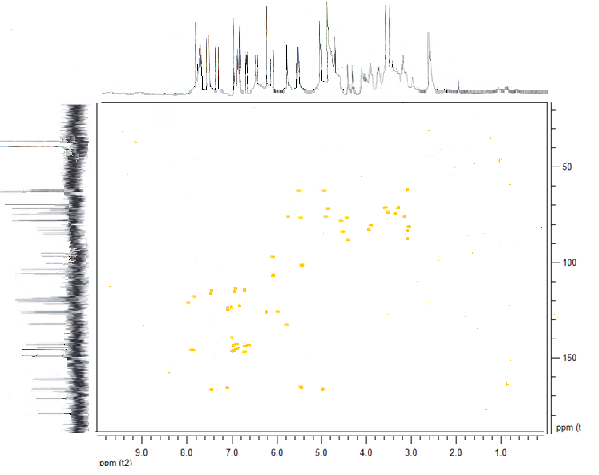


**Fig. S11**. HMBC spectrum of Compound 1.
